# Unraveling the Chemical Composition and Biological Activity of *Geum aleppicum* Jacq.: Insights from Plants Collected in Kazakhstan

**DOI:** 10.3390/molecules30193888

**Published:** 2025-09-26

**Authors:** Gulnur N. Kuntubek, Martyna Kasela, Kaldanay K. Kozhanova, Wirginia Kukula-Koch, Łukasz Świątek, Kinga Salwa, Piotr Okińczyc, Aleksandra Józefczyk, Jarosław Widelski, Gulnara M. Kadyrbayeva, Aigerim Z. Mukhamedsadykova, Zuriyadda B. Sakipova, Anna Malm

**Affiliations:** 1 Department of Engineering Disciplines of Good Practices, School of Pharmacy, Kazakh National Medical University, 88 Tole Bi Street, Almaty 050012, Kazakhstan; kuntubek.g@kaznmu.kz (G.N.K.); kadyrbayeva.g@kaznmu.kz (G.M.K.); mukhamedsadykova.a@kaznmu.kz (A.Z.M.); sakipova.z@kaznmu.kz (Z.B.S.); 2 Department of Pharmaceutical Microbiology, Medical University of Lublin, 1 Chodzki Street, 20-093 Lublin, Poland; martyna.kasela@umlub.edu.pl; 3 Department of Pharmacognosy with Medicinal Plant Garden, Medical University in Lublin, 1 Chodzki Street, 20-093 Lublin, Poland; wirginia.kukula-koch@umlub.edu.pl (W.K.-K.); aleksandra.jozefczyk@umlub.edu.pl (A.J.); jaroslaw.widelski@umlub.edu.pl (J.W.); 4 Department of Virology with Viral Diagnostics Laboratory, Medical University of Lublin, 1 Chodzki Street, 20-093 Lublin, Poland; lukasz.swiatek@umlub.edu.pl (Ł.Ś.); kinga.salwa@umlub.edu.pl (K.S.); 5 Department of Pharmacognosy and Herbal Medicines, Wrocław Medical University, 211a Borowska Street, 50-556 Wrocław, Poland; piotr.okinczyc@umw.edu.pl

**Keywords:** *Geum aleppicum* Jacq., yellow avens, chemical profile, bioactivity, antifungal activity, anticancer activity

## Abstract

*Geum aleppicum* Jacq. (yellow avens), a species traditionally used in folk medicine, remains understudied in the ethnopharmacological aspects. In this study, we comprehensively evaluated the phytochemical composition and biological activity of a hydroethanolic (50:50, *v*/*v*) extract from the aerial parts of *G. aleppicum* collected in Kazakhstan. Using the high-performance liquid chromatography coupled with electrospray ionization quadrupole time-of-flight tandem mass spectrometry (HPLC-ESI-QTOF-MS/MS), we identified 24 compounds, predominantly phenolic acids, flavonoids, tannins, and triterpenoids. The major compound was ellagic acid (2.28 mg/g dry extract) as revealed by the reverse phase high-performance liquid chromatography–diode array detector (RP-HPLC-DAD). The extract exhibited a high polyphenol content (131.45 mg GAE/g) and strong antioxidant activity in Ferric Reducing Antioxidant Power (FRAP) assay and 2,2-diphenyl-1-picrylhydrazyl (DPPH) radical scavenging assay (3.82 ± 0.07 mmol Fe^2+^/g and 106.61 ± 0.89 mg GAE/g, respectively). Antimicrobial assay of the extract revealed notable antifungal activity against *Candida* spp., especially against *C*. *glabrata* and *C*. *tropicalis* with minimum inhibitory concentration (MIC) of as low as 0.125 mg/mL, showing fungistatic effect. Although the extract inhibited the cytopathic effect induced by Human Herpesvirus 1 (HHV-1) in VERO cells, it did not significantly reduce viral replication. Moreover, among human cancer cell lines studied, the extract exerted moderate and selective cytotoxicity against A549 lung cancer cells (CC_50_ = 75.51 µg/mL, SI = 9). These findings highlight *G. aleppicum* as a rich source of bioactive compounds, especially phenolics, supporting its potential for development of pharmaceutical and cosmetic applications.

## 1. Introduction

The genus *Geum*, belonging to the Rosaceae family, is widely distributed across the temperate climate zones of Eurasia [1]. There are approximately 70 species within this genus, including both wild and cultivated perennial herbaceous plants. These species are a source of more than 300 compounds, such as monoterpenoids, sesquiterpenes, triterpenoids, flavonoids, steroids, hydrolyzable tannins, and phenylpropanoids. Many *Geum* species exhibit pharmacological activity, attributable to the abundance of these biologically active compounds [2,3]. Numerous species have well-documented medicinal properties, with most reports originating from Eastern Asia, including China, Japan, and Korea (e.g., *G. japonicum* L., *G. aleppicum* Jacq., *G. rivale* L.), but also from Russia (*G. rivale* L.), Iran (*G. iranicum* Khat., *G. kokanicum* Regel & Schmalh. ex Regel), and Europe (*G. rivale*, *G. urbanum* L.). These plants have proven useful in traditional medicine, primarily for the prevention and treatment of a variety of diseases, including gastrointestinal disorders (such as diarrhea, indigestion, and inflammation) as well as skin and mucous membranes conditions due to their antiseptic and anti-inflammatory properties [3]. Terpenoids and tannins are considered among the most active compounds produced by plants from the *Geum* genus. Accordingly, essential oils—especially rich in monoterpenoids and sesquiterpens—are often prepared from the roots and used as flavorings or cosmetic ingredients [4]. Multiple studies have also focused on the pharmacological profile of *Geum* extracts and demonstrated their wide range of properties, including angiogenic and cardioprotective activities (e.g., tannin and polyphenol fractions) [5,6], antihypertensive effect (tannins) [7], neuroprotective, and antidepressant properties (*G. japonicum* extract) [8,9] as well as antimicrobial, antitumor, anti-inflammatory activities, and many others [3]. Despite these reports, a little attention has been paid to *G. aleppicum*, another *Geum* species with a rich tradition of use in folk medicine; most of them investigate plant microbiome [10,11,12,13]. Only a few contain partial data on its chemical composition or biological activity [2,14,15,16,17,18], which leaves a gap that needs to be addressed.

*G. aleppicum* (yellow avens) thrives in valleys found in Japan, China, Mongolia, and Siberia region, as well as in Europe [15,19]. In Kazakhstan, this species is commonly encountered in mountainous regions ranging from the Altai to the Kungei Alatau. Although data on its pharmacobiological properties remain limited, reports indicate that *G. aleppicum* contains benzoic acid, gallic acid, salicylic acid, vanillin, 3,4,5-trihydroxybenzaldehyde, and 3,4,5-trihydroxybenzoic acid ethyl ester. Traditionally, it has been utilized as a diuretic and astringent [20]. While *Geum* species are known to be rich in phenolic compounds and extracts from these plants demonstrate diverse pharmaceutical activities for treating various diseases, comprehensive knowledge of their phytochemical composition is still lacking. Nevertheless, *G. aleppicum* is considered a promising source of secondary metabolites, making it an attractive candidate for applications in cosmetic and pharmaceutical industries [4].

The aim of our research was to analyze the chemical composition of hydroethanolic (50:50, *v*/*v*) extract obtained by ultrasonic-assisted method from the aerial parts of *G. aleppicum*. collected in Kazakhstan (further referred as *G. aleppicum* extract). The comprehensive characterization of its bioactivity was achieved by evaluating antibacterial, antifungal, antiviral, and anticancer activities, as well as antioxidant potential.

## 2. Results

### 2.1. Qualitative and Quantitative Composition of G. aleppicum Extract

The primary objective of this study was to elucidate the identities of the compounds present in the *G. aleppicum* extract. To achieve this, we employed liquid chromatography–mass spectrometry (LC-MS) and liquid chromatography–tandem mass spectrometry (LC-MS/MS) analyses, as the extract demonstrated significant pharmacological potential. Initially, the solvent gradient and mass spectrometer parameters were optimized to effectively separate the metabolites in the sample, facilitating the annotation of their individual constituents. The analyses were performed using an extract concentration of 10 mg/mL in 50% ethanol.

**Figure 1:**
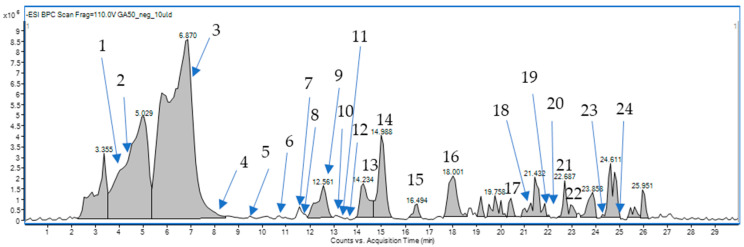
Mass chromatogram of the chemical composition of *G. aleppicum* extract.

Compound annotation was based on elemental composition, with a mass tolerance of 10 ppm. In total, more than 6000 signals with intensities exceeding 500 were detected in the total ion chromatograms (TICs) of the extract. However, given the focus on the biological properties of the sample, our analysis concentrated on the major components to ascertain their identities (Figure 1).

By analyzing retention times, accurate masses, fragmentation patterns, and the relevant scientific literature on the Rosaceae family, we were able to assign the structures of the 24 compounds tentatively. Their list is presented in Table 1 below and in the Supplementary Files (Table S1, Figure S1).

The phytochemical profile of the *G. aleppicum* extract revealed a diverse array of phenolic compounds, including phenolic acids, flavonoids, flavonoid glycosides, ellagic acid derivatives, and triterpenoids. Polyphenols from several structural groups were found to be the major constituents. Among the phenolic acids, gallic acid, protocatechuic acid, tachioside, caffeic acid, caffeoylmalic acid isomers, chlorogenic acid, ellagic acid, and hydroxybenzoic acid were identified, along with protocatechoylglucose. The flavonoid fraction comprised quercetin bis-hexuronide and tiliroside, while the extract also contained tannins such as pedunculagin I, pedunculagin II, trigalloyl hexose, and tellimagrandin I.

The content of selected compounds in *G. aleppicum* extract is presented in Table 2. The quantitative analysis showed that the major compound identified in the extract was ellagic acid (2.28 mg/g dry extract), followed by hydroxybenzoic acids, namely hydroxybenzoic acid (0.261 mg/g dry extract) and protocatechuic acid (0.215 mg/g dry extract). The reverse phase high-performance liquid chromatography–diode array detector (RP-HPLC/DAD) analysis also revealed a relatively high concentration of caffeic acid derivative corresponding to caffeoylmalic acid isomer in qualitative analysis (0.169 mg/g dry extract) and kaempferol derivative (0.14 mg/g dry extract). Chromatograms obtained with the use of high-performance liquid chromatography–photodiode array detection method (RP-HPLC/PDA) are presented in Figure S2 in the Supplementary Files.

### 2.2. Total Polyphenol Content, Total Flavonoid Content, and Antioxidant Activity

Colorimetric assays indicated that *G. aleppicum* extract is rich in polyphenols (131.45 ± 1.84 mg GAE/g (milligrams of gallic acid equivalents per gram of dry extract)), contains a moderate concentration of flavonoids (12.75 ± 0.17 mg QUE/g (milligrams of quercetin equivalents per gram of dry extract)), and exhibits significant antioxidant potential as measured by both the Ferric Reducing Antioxidant Power Assay (FRAP; 3.82 ± 0.07 mmol Fe^2+^/g) and 2,2-diphenyl-1-picrylhydrazyl (DPPH) radical scavenging assay (106.61 ± 0.89 mg GAE/g) (Table 3).

### 2.3. Antibacterial and Antifungal Activity

Data on antimicrobial activity of *G. aleppicum* extract obtained using the microbroth dilution method are presented in Table 4. The activity towards bacterial species studied was characterized by minimum inhibitory concentration (MIC) range of 2–8 mg/mL; the lowest MIC was noted for Gram-positive *S. aureus* and Gram-negative *P. aeruginosa* (2 mg/mL) and the highest (8 mg/mL) for *E. coli*. For all bacterial isolates, minimum bactericidal concentration (MBC) was determined. MBC/MIC ratios were below or equal to 4, suggesting the tested extract’s bactericidal properties.

The initial results of antifungal activity showed that *G. aleppicum* extract exhibited MIC against *C. albicans* of 1 mg/mL, exerting fungistatic effect (MFC/MIC = 8). The expanded analysis of multiple *Candida* reference strains showed that *C. glabrata* and *C. tropicalis* were the most susceptible to the extract (MIC = 0.125 mg/mL) but had the highest MFC values, resulting in a high MFC-to-MIC ratio equal to 64, which proved its strong fungistatic action. The extract inhibited other *Candida* reference strains with MIC equal to 1 mg/mL, exerting fungistatic (MFC/MIC = 8) or fungicidal effect (MFC/MIC = 4), depending on the species. In contrast, *C. auris* was found to be more insensitive to the extract as was revealed by an eight times higher MIC value (8 mg/mL). However, fungicidal activity was observed for this yeast species.

**Figure 2:**
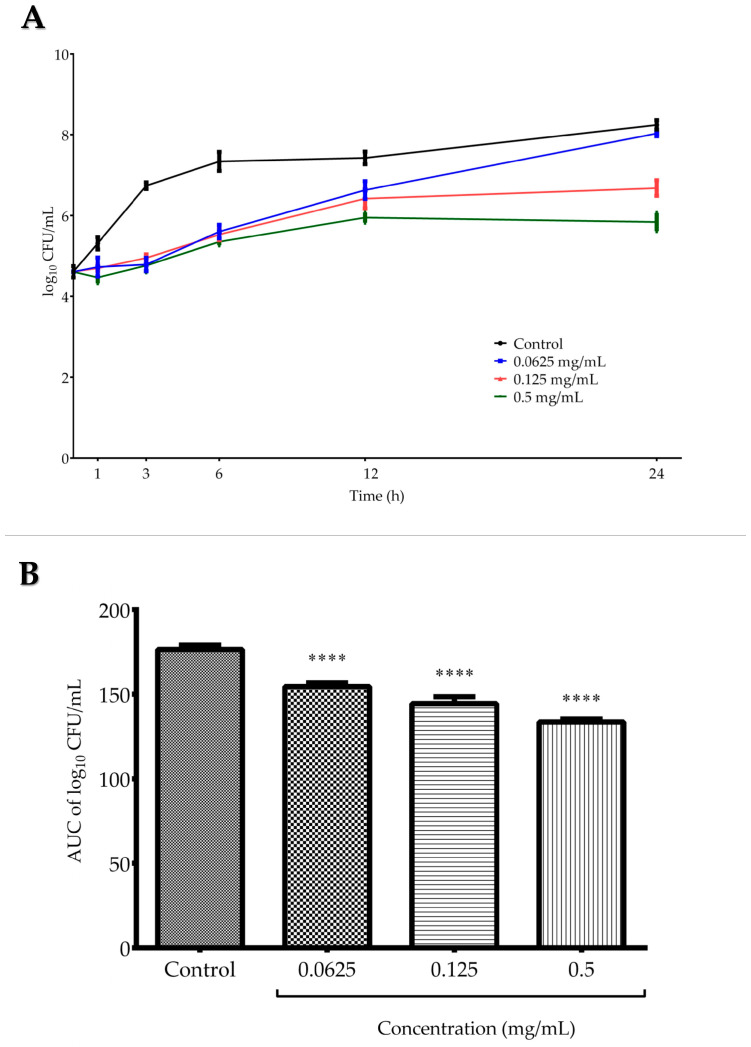
(**A**) Time–kill curves of *G. aleppicum* extract against *Candida tropicalis* ATCC 13803. (**B**) Time–kill kinetics based on the area under the curves (AUCs) of *G. aleppicum* extract against *C. tropicalis* ATCC 13803. Values are expressed as mean ± standard deviation based on three independent replicates. **** *p* < 0.0001 compared to the control (one-way ANOVA followed by Dunnett’s post hoc test).

Time–kill assay was conducted against *C. tropicalis* ATCC 13803 and included testing three *G. aleppicum* extract concentrations—0.0625, 0.125, and 0.5 mg/mL (Figure 2A). For all concentrations, there was a similar rate of inhibition in the viable number of fungal cells at the beginning of the incubation process (up to 6 h). Then, in the case of the lowest tested concentration, the fungal cells began to grow gradually, reaching a level comparable to that of the growth control after 24 h. Both in the medium and the highest applied concentration, the fungal growth remained stable after 12 h of incubation. At the final incubation point, the log_10_ CFU (colony forming units)/mL difference between 0.125 mg/mL (1×MIC) and the growth control was 1.56, confirming the fungistatic effect of the extract against *C. tropicalis*, i.e., the inhibition of cell proliferation. Figure 2B presents the analysis of area under the curves (AUCs) of time–kill kinetics of *G. aleppicum* extract, where the fungistatic activity of the extract was confirmed as a statistically significant decrease in the number of viable *C. tropicalis* cells in all tested concentrations when compared with the growth control after 24 h (*p* < 0.001).

### 2.4. Antiviral Activity

**Figure 3:**
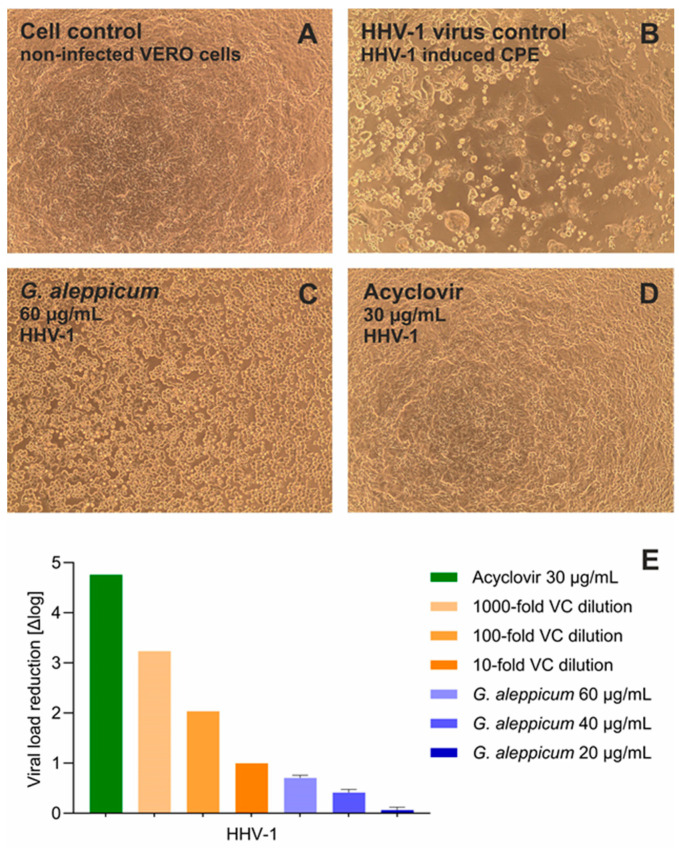
Antiviral potential of *G. aleppicum* extract against Human Herpesvirus 1 (HHV-1): (**A**) VERO cell monolayer, cell control; (**B**) HHV-1-induced cytopathic effect; HHV-1, virus control; (**C**) influence of *G. aleppicum* extract 60 µg/mL on HHV-1-infected VERO cells; (**D**) influence of acyclovir 30 µg/mL on HHV-1-infected VERO cells; (**E**) reduction in HHV-1 viral load in relation to virus control.

In this research, the antiviral potential of *G. aleppicum* extract was assessed against Human Herpesvirus Type 1 (HHV-1), also known as Herpes Simplex Virus 1 (HSV-1), and Human Coxsackievirus B3 (CVB3), representing DNA and RNA viruses, respectively. The cytotoxicity evaluation of *G. aleppicum* extract revealed that the 10% cytotoxic concentration (CC_10_), concentration decreasing the cellular viability by 10%, against the non-cancer monkey kidney cells (VERO) was 63.22 ± 4.82 µg/mL after 72 h incubation. Thus, the highest nontoxic concentration of the extract, being 60 µg/mL, was selected for the assessment of antiviral activity. The monolayer of VERO cells after 72 h incubation is presented in Figure 3A. Infection with HHV-1 resulted in the development of viral-induced cytopathic effect (CPE), observed as the disruption of the cell monolayer and intense cell rounding, which is shown in Figure 3B. During the first stage of screening for potential antiviral activity, the ability of tested samples to inhibit the CPE is assessed. *G. aleppicum* extract exerted a noticeable inhibition of the virus-induced CPE development, observed as the higher cell density (Figure 3C) than that in the virus control (Figure 3B). Acyclovir, a reference antiviral drug used in the treatment of HHV-1-associated infections, fully inhibited the development of CPE in the virus-infected VERO cells (Figure 3D). Subsequently, the influence of the tested extract and acyclovir on the replication of viral DNA was assessed. The qPCR amplification revealed that *G. aleppicum* extract at the concentrations of 60, 40, and 20 µg/mL reduced the HHV-1 viral load by 0.71, 0.41, and 0.07 log, respectively, compared to the HHV-1 virus control. At the same time, acyclovir reduced HHV-1 viral load by 4.76 log (Figure 3E).

Antiviral potential of *G. aleppicum* extract against CVB3 is presented in Figure S3 in Supplementary Files. The results demonstrated that CVB3 also induced CPE in VERO, but the incubation of the virus-infected VERO cells with *G. aleppicum* extract did not affect its development. Subsequent RT-qPCR analysis of RNA isolated from samples collected during the anti-CVB3 assay revealed that the viral load was comparable to the CVB3 virus control (Δlog ranging from −0.11 to 0.17).

### 2.5. Anticancer Activity

**Figure 4:**
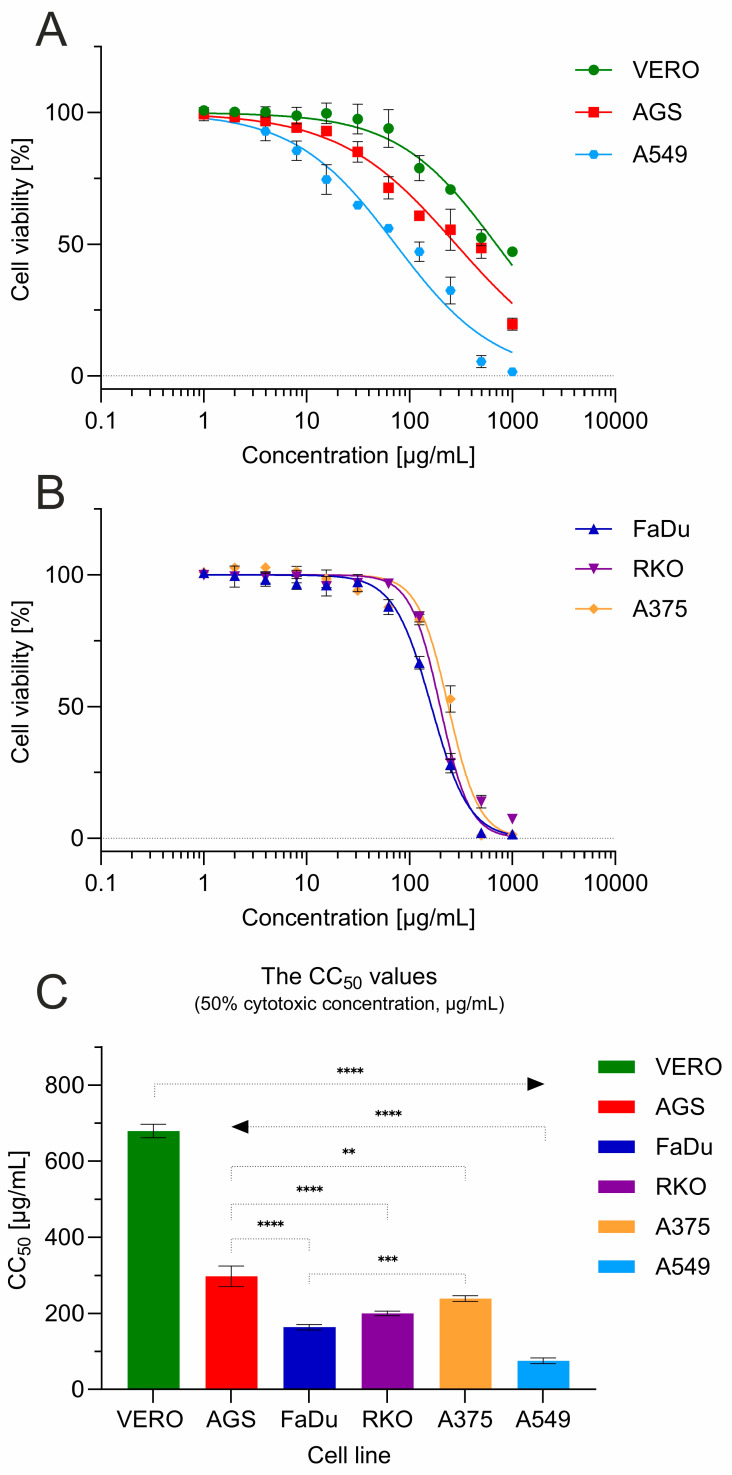
Cytotoxicity of *G. aleppicum* extract towards a panel of cell lines: (**A**) cell viability for VERO, AGS and A549; (**B**) cell viability for FaDu, RKO and A375; (**C**) comparison of CC_50_ values between cell lines (statistical significance: **** *p* < 0.0001, *** *p* < 0.001, ** *p* < 0.01).

The cytotoxic influence of *G. aleppicum* extract was assessed towards a panel of the following cell lines: VERO (non-cancer monkey kidney cells), RKO (human colon cancer cells), FaDu (human hypopharyngeal squamous-cell carcinoma cells), AGS (human gastric adenocarcinoma cells), A549 (lung cancer cells), and A375 (melanoma cells) (Figure 4).

The 50% cytotoxic concentration (CC_50_) against VERO cells, representing non-cancer cells, was 679.43 ± 17.86 µg/mL (Figure 4C). According to the classification of plant extract cytotoxicity described in the literature [26,27], this indicated the lack of significant cytotoxicity (CC_50_ > 500 µg/mL). Interestingly, *G. aleppicum* extract dose dependently decreased the viability of cancer-derived cells (Figure 4A,B), exerting significantly (*p* < 0.001) higher cytotoxicity than towards VERO cells. This cytotoxic effect was the most profound towards A549 cells originating from lung cancer, where the calculated CC_50_ was 75.51 ± 7.41 µg/mL, indicating a moderate (CC_50_ within the range of 20–200 µg/mL) and selective (SI = 9) cytotoxicity. A weak cytotoxic effect (CC_50_ within the range of 200–500 µg/mL) was observed against AGS cells, with a CC_50_ of 297.43 ± 27.05 µg/mL (SI = 2.28). When tested against other cancer cell lines FaDu, RKO, and A375, *G. aleppicum* extract showed a similar course of dose–response curves (Figure 4B), with CC_50_ values of 163.6, 199.73, and 239.05 µg/mL, respectively, and SI ranging from 2.84 to 4.15.

## 3. Discussion

The analysis showed that hydroethanolic *G. aleppicum* extract obtained by ultrasonic-assisted extraction was characterized by a substantial presence of phenolic compounds recognized for their strong antioxidant properties, which suggests significant potential for health-promoting and pharmacological applications. We observed high content of polyphenols in our extract (131.45 ± 1.84 mg GAE/g) and the presence of ellagic acid glucoside, which further enhanced the extract’s profile, indicating its potential utility in therapeutic applications related to oxidative stress [28,29].

The flavonoid constituents identified include quercetin bis-hexuronide and tiliroside proven to show anti-inflammatory, antioxidant, and antimicrobial effects [30]. Also, the extract contained various tannins (pedunculagin I, pedunculagin II, trigalloyl hexose, and tellimagrandin 1), recognized for their astringent properties and contributes to the extract’s potential health benefits, particularly in managing inflammation and microbial infections [31].

The tentative assignment of polyphenols in the investigated *G. aleppicum* extract is in accordance with the former studies on *G. rivale* by Orlova et al. [22]. Their research emphasizes the identification and biological activity of the major phenolic constituents within the plant, which include various derivatives of phenolic acids, flavonoids, and tannins. Key compounds identified in this species include quercetin, kaempferol, isorhamnetin and their derivatives, ellagic acid and its derivatives, or pedunculagin that support the extract’s antibacterial, antioxidant, and neuroprotective activities.

Among the 24 constituents identified in *G. aleppicum* extract in the present study, the terpenoids take a special place. Tormentic acid, hydroxytormentic acid isomers, trihydroxyursendioic acid, geumonid, and pomolic acid constituted a substantial contribution to the overall phytochemical profile of the crude plant extract. Notably, these constituents have been previously reported in *G. japonicum*, a species native to East Asia, where their therapeutic properties have been experimentally validated [25].

The pharmacological relevance of triterpenoids is related to a potent antiplatelet activity primarily through the upregulation of cyclic guanosine monophosphate (cGMP). This signaling pathway is crucial for maintaining vascular integrity and preventing thrombus formation, which can lead to ischemic events. Specifically, the elevation in cGMP inhibits the activation of integrin αIIbβ3, a key player in platelet aggregation, thereby reducing the risk of thrombus formation. In addition to their antiplatelet effects, tormentic acid has demonstrated anti-inflammatory and antioxidant activities, which can further contribute to cardiovascular protection and antidiabetic action by mitigating oxidative stress and inflammation—two critical factors in the pathogenesis of atherosclerosis [25,32,33].

Quantitative analysis showed that ellagic acid (2.28 mg/g dry extract) was the main compound in the analyzed *G. aleppicum* extracts. Other studies have shown that ellagic acid and its derivatives content in *G. aleppicum* extract may change depending on the time that the plant was harvested. Kashchenko et al. (2023) [16] observed that in methanolic (70%) extract from the aerial parts of *G. aleppicum* collected in Russia, the content of ellagic acid was the highest in the fruiting phase, during September (5.63 mg/g dry extract) in comparison to May (traces) or July (0.89 mg/g dry extract). On the other hand, the authors detected numerous ellagic acid derivatives, not detected in this study, which may be connected to plant metabolism, where ellagic acid is being produced as a result of hydrolysis of ellagitannins dominating during flowering stages at spring [16].

Our study demonstrated for the first time that *G. aleppicum* extract possessed substantial antioxidant potential, as evidenced by FRAP (3.82 ± 0.07 mmol Fe^2+^/g) and DPPH (106.61 ± 0.89 mg GAE/g) assays. Due to limited research on this species and considerable methodological discrepancies across studies, direct comparisons with the existing literature remain challenging. However, our findings confirm the notable antioxidant capacity of *G. aleppicum* extract. Previous investigations have examined the antioxidant properties of underground plant parts, highlighting their therapeutic potential. For example, Owczarek et al. found that hydroethanolic extract from aerial parts of *G. rivale* collected in Poland exhibited moderately lower antioxidant capacity compared to underground parts (2.23 mmol Fe^2+^/g vs. 3.84 mmol Fe^2+^/g) [21]. This pattern was consistent with their DPPH assay results, where aerial parts showed 78.3 mg GAE/g compared to 174.8 mg GAE/g for underground parts. The same team investigated the total polyphenol content in the hydroethanolic extract from *G. rivale* and *G. urbanum*, which in their case was lower (78.3 and 78.9 mg GAE/g, respectively) than in our *G. aleppicum* extract (131.45 mg GAE/g).

Studies conducted herein demonstrated for the first time that *G. aleppicum* extract exhibited antibacterial activity within a comparable MIC range (2–8 mg/mL) and displayed a broader spectrum of antifungal activity, with MIC values for reference *Candida* species ranging from 0.125 to 8 mg/m. It is worth noting that the lowest MIC values (0.125 mg/mL) were found for *C. glabrata* (now classified to *Nakaseomyces* genus) and *C. tropicalis*. Notably, the extract displayed distinct modes of action against bacteria and yeasts. For bacteria, the ratio of MBC to MIC ranged from 1 to 4, indicating a bactericidal effect. In contrast, for fungi, the ratio of MFC to MIC suggested a predominantly fungistatic effect, a finding further supported by the time–kill assay performed with *C. tropicalis* where the proliferation of fungal cells was achieved in all tested concentrations when compared with the growth control (*p* < 0.001). Moreover, both species—*C. glabrata* and *C. tropicalis*—which were the most sensitive to *G. aleppicum* extract have been increasingly reported to colonize the human body, enhancing their significance as etiological agents of candidiasis, including invasive diseases. Consequently, these two yeast species have been listed by the WHO as priority fungal pathogens [34,35].

Numerous compounds identified in *G. aleppicum* extract in our study demonstrate significant potential as antifungal agents against *Candida* species. Gallic acid demonstrates the strongest and most well-documented broad-spectrum effectiveness against various *Candida* species (<0.156 mg/mL), most likely due to the disruption of cell membrane structure [36]. Also, ellagic acid exhibits potent antifungal activity, not only having low MIC values against clinical *C. albicans* isolates in the range of 0.25–2 mg/mL but also increasing the activity of fluconazole, reducing its MIC values up to 32-fold when used in combination [37]. Similarly, tellimagrandin I and caffeic acid act synergistically with conventional antifungals [38,39]. Moreover, *C. glabrata*, especially susceptible to *G. aleppicum* extract in this study, is well known for inherently reducing susceptibility to azole antifungal drugs, which are frequently used in both the treatment and prophylaxis of candidiasis. This reduced susceptibility is clinically significant, as it limits the effectiveness of commonly prescribed azole agents such as fluconazole and itraconazole [40]. As demonstrated by the above-mentioned studies, many of these compounds show particular promise when used in combination with conventional antifungals, suggesting potential applications in combating drug-resistant *Candida* infections. Nevertheless, most studies are currently limited to in vitro *conditions*, thus further clinical research is needed to establish safety profiles for human use and possible therapeutic applications.

*G. aleppicum* extract in our study exhibited fungistatic activity. Fungistatics are widely used in medicine, offering significant advantages over fungicidal agents. These benefits are particularly evident in antifungal therapies for prophylactic treatment in immunocompromised patients or in the management of chronic infections, where they result in a lower risk of inflammatory response [41,42]. Plant extracts with fungistatic activity also hold promise for applications in cosmetology. They are incorporated into shampoos, creams, and other products designed for the prevention of fungal infections or as supportive agents in topical formulations for the treatment of fungal infections [43,44].

The studies on the antibacterial and antifungal activities of *Geum* species are notably constrained, with most investigations relying predominantly on qualitative methodologies. For instance, Hee Ham et al. (2022) employed the well-diffusion assay to evaluate the antibacterial properties of methanol extracts derived from *G. aleppicum* [2]. However, their findings did not demonstrate significant antibacterial activity. Furthermore, the study exhibited methodological limitations, such as the omission of reference microorganism strains and the absence of positive controls (e.g., standard antibiotics), which undermine the credibility and usefulness of the results. Similarly, other researchers have utilized diffusion-based approaches, restricting their assessments to only two clinical strains—*E. coli* and *S. aureus*. That study reported marginally greater antibacterial activity against the Gram-negative *E. coli* compared to Gram-positive *S. aureus* as revealed by zone of growth inhibition of 16 mm and of 11 mm, respectively. Nevertheless, the limited scope and qualitative nature of these investigations highlight the need for more comprehensive and quantitatively rigorous research in this area.

Human herpesvirus 1 (HHV-1), formerly referred to as Herpes simplex virus 1 (HSV-1), belongs to the Herpesviridae family. It is a human neurotropic, enveloped, double-stranded DNA virus. A characteristic feature shared by members of the Herpesviridae family is their capacity to evade the immune response while maintaining latent infections with minimal gene transcription. This latency is established in sensory neurons and ganglia, predominantly in the trigeminal and sacral, and persists throughout the host’s lifetime. Typically, HHV-1 is responsible for oral cold sores and keratitis of the eyes. However, it can also induce severe, life-threatening conditions in immunocompromised individuals, including neonates, AIDS patients, and individuals undergoing immunosuppressive therapy. Among antiviral agents, acyclovir is considered the first-line treatment due to its efficacy. Unfortunately, extended antiviral therapy has contributed to the emergence of drug-resistant HHV-1 mutants [45,46]. Coxsackievirus B3 (CVB3) is a cardiovirulent enterovirus classified within the family Picornaviridae, which may be implicated in myocarditis and dilated cardiomyopathy (DCM). Unlike herpesviral infections, therapeutic options for antiviral treatment of CVB3-associated myocarditis remain limited. Pleconaril and ribavirin have demonstrated efficacy in inhibiting CVB3 replication, thereby reducing myocardial damage. Nevertheless, no specific antiviral treatment for CVB3 infections is currently available [47]. As drug-resistant mutants of HSV-1 emerge and there is no specific antiviral treatment for CVB3 infections, the need for research on new antiviral drugs is clear. A successful antiviral drug candidate is expected to reduce the viral load or infectious titer by at least 3 log [48]. Thus, despite the noticeable inhibition of CPE in virus-infected VERO cells together with cytolytic influence of HHV-1, *G. aleppicum* extract did not significantly decrease the viral loads of HHV-1 (reduction of 0.71 log at the highest tested concentration of 60 µg/mL). Therefore, no antiviral activity has been reported for this plant under experimental conditions used. However, in certain circumstances, the inhibition of CPE in virus-infected cells without significant reduction in viral replication may be a desirable mode of action in drugs supporting the treatment of viral infections. By modulating host cell pathways, these drugs can act as cell-protective agents, inhibiting apoptosis or inflammation, limiting organ damage during infection, and preventing disease symptoms [49]. For these reasons, further studies may also focus on identifying compounds in *G. aleppicum* responsible for such cytoprotective effect.

Similar to reports on the antibacterial and antifungal activity of the *Geum* genus, studies on its antiviral activity are not only few in number but also limited in terms of applied methodology, resulting in multiple limitations. For example, both ethyl acetate and n-butanol *G. urbanum* extracts were reported by Zaharieva et al. to have antiviral activity against human adenovirus type 5 and HHV-1. However, the antiviral assays included in this research were based only on the assessment of the viability of virus-infected cells, without testing the impact on actual viral replication, like viral titer or viral load reduction [23]. Our research measured the impact on viral replication within the infected cells and proved that there was no significant reduction in viral load, as measured based on amplification of viral nucleic acids. Most of the studies on antiviral activity of *Geum* genus focus on *G. japonicum*. The infusion prepared from *G. japonicum* and isolated eugenin were shown to exert anti-herpesviral activity in plaque reduction assays, and further molecular studies revealed their ability to inhibit viral DNA polymerase activity as the underlying mechanism [50,51]. Interestingly, *G. japonicum* was also reported to inhibit another representative of human herpesviruses, the cytomegalovirus (CMV) known also as Human Herpesvirus–5 (HHV-5) [52]. *G. japonicum* was also shown to exert synergism with acyclovir, both in vitro and in vivo. Female BALB/c mice infected with HHV-1 and treated with acyclovir and *G. japonicum* extract showed a greater reduction in virus yield in the brain compared to when treated with acyclovir alone [53]. The methanolic extract from *G. japonicum*, as well as isolated ursolic acid, maslinic acid, and geumonoid, were found to inhibit the protease of human immunodeficiency virus (HIV-1) [54,55].

Among anticancer properties investigated by other authors, the in vitro antineoplastic of *G. urbanum* L. revealed that the ethyl acetate extract obtained from aerial parts of this plant exerted the highest cytotoxicity towards cells from bladder cancer with low CC_50_ (21.33 µg/mL) but relatively low selectivity (SI < 3) when compared to the effect on non-cancerous human kidney cells (HEK293). Importantly, this extract showed overall moderate cytotoxicity to HEK293 (CC_50_ = 62.30 µg/mL), which raises safety concerns about its practical application [23]. For this research, we have selected cell lines originating from various cancers, including head and neck cancers represented by FaDu, cancers of the gastrointestinal tract (AGS and RKO), as well as lung (A549) and skin (A375) carcinomas. Taken together, these cancer types represent almost 35% of all cancers diagnosed in 2022 [56]. *G. aleppicum* extract investigated in the present study exhibited moderate and selective cytotoxicity against A549 lung cancer cells (CC_50_ = 75.51 µg/mL, SI = 9). Notably, according to the GLOBOCAN estimates produced by the International Agency for Research on Cancer (IARC), lung cancer was the most frequently diagnosed cancer in 2022 [56].

Based on the performed fingerprinting of *G. aleppicum* metabolites and the results of biological activity, this plant stands out as a bioactive-rich herb with significant implications for therapeutic properties, particularly due to its high content of phenolic compounds that can exhibit a wide variety of pharmacological actions.

## 4. Materials and Methods

### 4.1. Plant Collection and Extract Preparation

**Figure 5:**
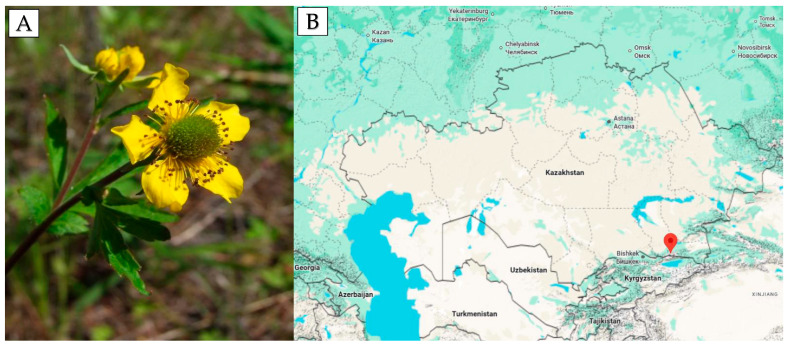
(**A**) *Geum aleppicum* Jacq. during the flowering stage; (**B**) geographical location of the habitat where the plant material was collected.

The aerial parts of *G. aleppicum* Jacq. were collected in May 2021 during the flowering stage in their natural habitat near the Zailiysky Alatau mountains (Almaty, Kazakhstan; 43°10′45.3″ N 77°05′37.6″ E) (Figure 5A,B). Over 5 kg of raw material was gathered for plant identification and standardization. For the extraction experiment, 25 g of crushed raw material was used. The plant identification was carried out following the State Pharmacopeia of the Republic of Kazakhstan by Gulnara Tokbergenovna Sitpaeva at the Laboratory of Higher Plant Flora of the Institute of Botany and Phytointroduction of the Committee of Forestry and Wildlife of the Ministry of Ecology, Geology, and Natural Resources of Kazakhstan (Voucher number 01–05/307). After collection, all impurities such as other plants and soil were removed in compliance with the Good Agricultural and Collection Practice guidelines. The extraction process was conducted according to the method described in the patent by [57]. The plant material was air-dried under a canopy in natural conditions at a temperature of 30–35 °C for several days and then shredded. Voucher specimens were deposited in the Institute of Botany and Phytointroduction to replenish the herbarium fund of the main botanical garden.

For the extraction, the aerial parts of the plant, consisting of flowers, leaves, and stems, were cut into 3–4 cm pieces. Then, 300 mL of 50% ethanol (*v*/*v*) was added to 25 g of the crushed raw material placed in a 500 mL flask, and the mixture was processed in an ultrasonic extractor (KQ5200B; Kunshan Instruments Inc., Kunshan, China) for 25 min (room temperature, 40 kHz). The process was repeated three times with the same solvent. To obtain the dry extract, the solvent was evaporated at 45°C for 8 h (Concentrator plus, Eppendorf, Hamburg, Germany). The extraction yield was 24.93 ± 0.28 g per 100 g of dry weight.

### 4.2. The HPLC-ESI-QTOF-MS/MS Fingerprinting

The high-performance liquid chromatography coupled with electrospray ionization quadrupole time-of-flight tandem mass spectrometry (HPLC-ESI-QTOF-MS/MS) was employed for the fingerprinting of the samples in a manner described previously [58]. The experimental setup comprised a sophisticated instrument featuring a degasser, a binary solvent pump, a peristaltic pump for reference ions, an autosampler, a UV detector, and a Q-TOF mass spectrometer equipped with an electrospray ionization source. Specifically, the Agilent Technologies (Santa Clara, CA, USA) HPLC-ESI-Q-TOF-MS platform was utilized, which included an HPLC chromatograph (1200 series) integrated with a Zorbax Eclipse Plus RP-18 chromatographic column (150 mm × 2.1 mm; dp = 3.5 µm). The system components encompassed a degasser (G1322A), a binary pump (G1312C), an autosampler (G1329B), a photodiode array detector (DAD, G1315D), and a mass spectrometer (G6530B). For the acquisition of mass spectral data and subsequent processing, Agilent MassHunter workstation software (version B.10.00) was employed (Santa Clara, CA, USA).

A solvent gradient was implemented, comprising solvent A (water with 0.1% formic acid) and solvent B (acetonitrile with 0.1% formic acid) as follows: 0.0 min | 1% B, 10 min | 20% B, 15 min | 60% B, 17–22 min | 95% B, and 22.1 min | 1% B. The total duration of the analysis was 30 min, with an injection volume of 10 µL, a flow rate of 0.2 mL/min, and a column temperature maintained at 20°C.

Compound identification was achieved through high-resolution mass measurements, analysis of MS/MS fragmentation patterns, and the integration of previously published data regarding the composition of the specific plant species under investigation. The mass spectrometer was operated under the following settings: *m*/*z* range of 100–1400 Da, gas and sheath gas temperatures at 275°C and 325°C, respectively, gas flows of 12 L/min, fragmentor voltage of 110 V, skimmer voltage of 65 V, capillary voltage of 3000 V, and collision energies of 10 and 20 V.

### 4.3. RP-HPLC/DAD Analysis

For quantitative and qualitative determinations, the HPLC method utilized 27.62 mg of the *G. aleppicum* extract, which was dissolved with the aid of ultrasound in 2 mL of a 1:1 (*v*/*v*) ethanol–water mixture. This mixture was then purified of potential chlorophyll residues using solid-phase extraction (SPE) and 500 mg C18 micro-columns (J.T. Baker, Phillipsburg, NJ, USA). After column activation, the extract (2 mL) applied to the column was eluted with a 50% ethanol–water mixture [59]. Finally, a 10 mL extract was obtained, which was analyzed by RP-HPLC using a Zorbax Eclipse XDB C8 column (150 X 4.6 mm I.D., dp = 5 μm) as the solid phase in a gradient development system allowing for good separation of polyphenolic components: A—water + 1% acetic acid; B—acetonitryle (0.0 min, 10% B | 0–10 min, 10–14% B | 10–25 min, 14–30% B | 25–35 min, 30–35% B | 35–50 min, 35–90% B | 50–55 min, 90% B), flow–1 cm^3^/min, and column thermostat temperature–250°C. The identification of the compounds from HPLC analysis was performed by comparing retention times with those for standard solutions, spectroscopically determining their spectra in UV (λ = 254, 280 and 325 nm). The content of derivative compounds was calculated for specific compounds, e.g., chlorogenic acid derivative for chlorogenic acid, etc., as described in detail previously [60]. The content of components was calculated using results that took into account the wavelength at which the result was highest for a given compound. Collected chromatographic and spectroscopic data (peak areas corresponding to the concentrations of each quantified compound or derivative) were collected for inter-day (*n* = 3) analyses of both reference substance solutions and samples of *G. aleppicum* extract. The repeatability of peak areas was determined by the evaluation of both SD and RSD parameters. Standard substances were purchased from Sigma-Aldrich^®^ Co. (Livonia, MI, USA) and ChromaDex Inc. (Los Angeles, CA, USA).

### 4.4. Total Polyphenol Content and Total Flavonoid Content Evaluation

To determine the total polyphenol content, total flavonoid content, and antioxidant activity (described in detail in the next chapter), the dry extract was diluted in a DMSO–methanol solution (5:95, *v*/*v*) to obtain a concentration of 1 mg of extract per 1 mL of solvent. The solution was then subjected to serial double dilutions ranging from 2- to 16-fold and used in all colorimetric assays.

The total polyphenol content was measured spectrophotometrically using the Folin–Ciocalteu assay [61]. Prior to analysis, 200 µL of the extract dilutions were mixed with 40 µL of Folin–Ciocalteu reagent. After 5 min, 800 µL of 100 g/L Na_2_CO_3_ solution was added and mixed. Following 50 min of incubation at room temperature in the dark, the absorbance was read against a blank (prepared similarly using pure solvent instead of the sample) at 725 nm in disposable polystyrene 96-well plates (FL Medical, Torreglia, Italy) using an absorbance microplate reader (Agilent™ BioTek Epoch 2 Microplate Spectrophotometer; Agilent, Santa Clara, CA, USA). Results were calculated using freshly prepared gallic acid standard solutions (2.5–100 µg/mL) and expressed as milligrams of gallic acid equivalents per gram of dry extract (mg GAE/g extract). The standard deviation of the measurements was below 5%.

The total flavonoid content was determined spectrophotometrically using a modified pharmacopeial method with AlCl_3_ [61]. An aliquot of 50 µL of the extract dilutions was mixed with 50 µL of 2% ethanolic AlCl_3_ solution (*w*/*v*) and incubated for 60 min at room temperature in the dark. The absorbance was then measured at 420 nm using the same microplate reader. Results were calculated using freshly prepared quercetin standard solutions (2–400 µg/mL) and expressed as mg QUE/g. All measurements were performed in triplicate. The standard deviation of the measurements was below 5%.

### 4.5. Antioxidant Activity Evaluation

The FRAP assay was performed as described previously [56]. In this assay, the reagent was prepared by adding 10 mmol/L TPTZ (2,4,6-tris(2-pyridyl)-s-triazine) to 20 mmol/L ferric chloride in acetate buffer (pH 3.6).

Prior to the analysis, 20 μL of the extract dilutions was mixed with 200 μL of the ferric–TPTZ reagent and incubated for 4 min at room temperature in the dark. The assay was conducted in 96-well plates (FL Medical, Torreglia, Italy), and absorbance was measured at 593 nm using an absorbance microplate reader (Agilent™ BioTek Epoch 2 Microplate Spectrophotometer; Agilent, Santa Clara, CA, USA). Results were calculated using a calibration curve prepared with ferrous sulfate (0.02–1.5 μmol/mL) and expressed as mmol Fe^2+^ equivalents per gram of dry extract (mmol Fe^2+^/g extract). The standard deviation of measurements was below 5%.

In the DPPH assay, 20 μL of the diluted test extracts was mixed with 200 μL of 0.315 mM DPPH solution in methanol and incubated for 30 min at room temperature in the dark. The assay was carried out in 96-well plates, and absorbance was recorded at 517 nm using the same microplate reader. Results of the DPPH assay were expressed as mg GAE/g extract. All measurements were performed in triplicate, with a standard deviation below 5%.

### 4.6. Microbroth Dilution Method

The antimicrobial properties of *G. aleppicum* extract were determined by microbroth dilution method in a way described previously in the concentration range of 16–0.0156 mg/mL [62]. Parameters MIC, MBC, or MFC were determined against Gram-positive *Staphylococcus aureus* ATCC 29213, Gram-negative bacteria–*Escherichia coli* ATCC 25922, and *Pseudomonas aeruginosa* ATCC 27853 as well as fungi, represented by yeast *Candida albicans* ATCC 10231, and then against the panel of other non-*C. albicans* species based on the initial results—*C. krusei* ATCC 14243, *C. parapsilosis* ATCC 22019, *C. tropicalis* ATCC 13803, *C. glabrata* ATCC 90030, *C. lusitaniae* ATCC 34449, and *C. auris* CDC B11903. Vancomycin, ciprofloxacin, and fluconazole were used as reference antimicrobial substances against Gram-positive bacteria, Gram-negative bacteria, and yeasts, respectively. Results are presented as a mode from three independent experiments.

### 4.7. Time–Kill Assay

The time–kill assay was conducted against *C. tropicalis* ATCC 13803 using three extract concentrations corresponding to the minimum inhibitory concentration (MIC, 0.125 mg/mL), a subinhibitory concentration (0.0625 mg/mL), and a concentration exceeding the MIC fourfold (0.5 mg/mL). The *C. tropicalis* reference strain was selected based on results from the microbroth dilution method and its increasing clinical relevance.

Briefly, a fresh fungal inoculum of approximately 10^4^ CFU/mL was incubated at 35 °C with shaking at 250 rpm for 24 h under the following conditions: without extract (control) and with the three specified extract concentrations. Viable fungal cell counts were enumerated after 1, 3, 6, 12, and 24 h of incubation. The assay was performed in triplicate, and results are presented as the mean ± standard deviation (SD) of the log_10_ CFU/mL. A difference of ≥3 log_10_ CFU/mL between the MIC-treated sample and the growth control was interpreted as fungicidal activity, while a difference of <3 log_10_ CFU/mL indicated fungistatic activity.

Graphical representation of the time–kill assay results was generated using GraphPad Prism (v. 6.0; GraphPad Software Inc., San Diego, CA, USA), and statistical analysis was performed using one-way ANOVA followed by Dunnett’s post hoc test. Statistical significance was set at *p* < 0.05.

### 4.8. Antiviral Activity Evaluation

The antiviral activity was evaluated against CVB3 (ATCC, VR-30) and HHV-1 (ATCC, VR-260). Both viruses were cultivated in VERO cells. The experiments followed previously published protocols [62,63]. VERO cells were seeded into 48-well plates and incubated overnight. Subsequently, the cells were infected with CVB3 or HHV-1 at a concentration of 100-fold CCID_50_ (50% cell culture infective dose)/mL. For control purposes, at least two wells per plate remained uninfected (cell control). After a 1 h incubation, the infected monolayers were washed with PBS to remove unbound virions, then treated with *G. aleppicum* extract at non-cytotoxic concentrations, which were hereby defined as the concentrations decreasing the cellular viability by no more than 10%. The plates were incubated until CPE appeared in the virus control (infected but untreated cells). An inverted microscope (CKX41, Olympus, Tokyo, Japan), equipped with a camera (Moticam 3+, Motic, Hong Kong), was used to observe and document the plates. The plates underwent three freeze–thaw cycles at −76°C, after which samples were taken to measure the viral load. Ribavirin at 500 µg/mL and acyclovir at 30 µg/mL were used as antiviral controls targeting CVB3 and HHV-1, respectively.

Samples obtained from anti-CVB3 tests underwent RNA extraction using the QIAamp Viral RNA Mini Kit (QIAGEN GmbH, Hilden, Germany). In contrast, DNA was extracted from samples taken during anti-HHV-1 experiments with the QIAamp DNA Mini Kit (QIAGEN GmbH). The RNA isolates were subjected to one-step RT-qPCR (reverse-transcription quantitative polymerase chain reaction) amplification using an iTaq Universal SYBR Green One-Step Kit (Bio-Rad Laboratories, Life Science Group, Hercules, CA, USA) and enterovirus-specific primers (entrinR (5′-GAAACACGGACACCCAAAGTA-3′) and entrinF (5′-CGGCCCCTGAATGCGGCTAA-3′)) on a CFX96 thermal cycler (Bio-Rad Laboratories). The DNA isolates from anti-HHV-1 assays were subjected to qPCR amplification with SsoAdvanced Universal SYBR Green Supermix (Bio-Rad Laboratories) and primers (UL54F–5′CGCCAAGAAAATTTCATCGAG 3′, UL54R–5′ ACATCTTGCACCACGCCAG 3′) on the CFX96 thermal cycler. The one-step RT-qPCR and qPCR amplification conditions adhered to the manufacturer’s instructions. The viral loads of CVB3 and HHV-1 in samples treated with ASEO were compared to virus control using the ΔCq method via CFX Manager™ Dx Software (version 3.1) from Bio-Rad Laboratories, and the viral load reduction rates were calculated. The sensitivity of RT-qPCR and qPCR techniques was tested by analyzing serial dilutions (10-fold, 100-fold, and 1000-fold) of virus RNA (for CVB3) and DNA (for HHV-1) isolates.

### 4.9. Anticancer Activity Evaluation

The cytotoxic effects of *G. aleppicum* extract were assessed using VERO cells (ATCC CCL-81) and, for comparison, against cancer-derived cells from colon (RKO; ATCC CRL-2577), hypopharynx (FaDu; ATCC HTB-43), stomach (AGS; ATCC CRL-1739), lung (A549; ATCC CCL-185), and skin (A375; ATCC CRL-1619). The VERO and A375 cells were cultured in Dulbecco’s Modified Eagle’s Medium (DMEM; Corning, Tewksbury, MA, USA), and A549 cells were cultured in DMEM/F12 (Dulbecco’s Modified Eagle’s Medium/Ham’s F-12 50/50 Mix; Corning, New York, NY, USA), while Minimum Essential Medium (MEM; Corning) was used for the remaining cell lines. Cell media were supplemented with antibiotics (Penicillin–Streptomycin Solution, 100×, Corning) and fetal bovine serum (FBS; Corning). The stock solution of *G. aleppicum* extract was prepared at 50 mg/mL in DMSO (cell culture grade, PanReac Applichem, Darmstadt, Germany) for use in cytotoxicity and antiviral testing.

To determine non-cytotoxic concentrations, the microculture tetrazolium (MTT) assay was employed [64]. This involved treating cell monolayers in 96-well plates with serial dilutions of the *G. aleppicum* extract stock solution for 72 h. After treatment, the media were removed, cells were washed with PBS, and MTT-supplemented medium was added. Following a 3 h incubation, the formazan product was dissolved, and overnight incubation was performed. Absorbance was then measured at 540 nm and 620 nm using the Synergy H1 Multi-Mode Microplate Reader (BioTek Instruments, Inc., Winooski, VT, USA). The data were analyzed with GraphPad Prism (version 10.2.0) to calculate CC_50_ values (50% cytotoxic concentration) from dose–response curves using non-linear regression. Based on the CC_50_ values, the selectivity indexes (SIs) were calculated (SI = CC_50_VERO/CC_50_Cancer) to assess the specificity towards cancer cell lines. The cytotoxicity results’ statistical significance was assessed using GraphPad Prism with a one-way ANOVA and Tukey’s post hoc test, considering *p* < 0.05 as statistically significant.

## 5. Conclusions

Although *Geum aleppicum* Jacq. can be considered a promising source of secondary bioactive metabolites, the literature data is limited regarding its ethnopharmacological aspects. The hydroethanolic extract obtained from aerial parts of this species growing in Kazakhstan was characterized by a substantial presence of phenolic compounds. In addition, flavonoids, tannins, and triterpenoids were also detected. In the present study, we provide novel evidence of the promising antifungal and anticancer activities of this extract, along with its cytoprotective effect against HHV-1-induced cytopathic changes, which together may serve as a foundation for further in-depth investigations.

## 6. Patents

Method for preparing extract from above-ground parts of *Geum aleppicum* Jacq. yellow avens plant (patent no. 8117, 26 January 2024). This process involved Kuntubek Gulnur Nurbolatovna, Mukhamedsadykova Aigerim Zhumagazievna, Kozhanova Kaldanay Karzhauovna, Mombekov Serzhan Yessimbayevich. The applicant and patent holder are as follows: Asfendiyarov Kazakh National Medical University, 2023/0059.2 and the application submission date was 24 January 2023.

## Figures and Tables

**Table 1:** The list of high-performance liquid chromatography coupled with electrospray ionization quadrupole time-of-flight tandem mass spectrometry (HPLC-ESI-QTOF-MS/MS) tentatively identified components in the *G. aleppicum* extract.

No.	Ion	Rt (min)	Formula	*m*/*z* (calc.)	*m*/*z* (exp.)	Δ (mmu)	RDB	MS/MS Fragments	Compound	Ref.
**1**	[M-H]^-^	4.158	C_13_H_16_O_9_	315.0722	315.0739	−5.52	6	153	Protocatechoylglucose	[21]
**2**	[M-H]^-^	4.359	C_7_H_6_O_5_	169.0137	169.0159	2.2	5	125, 79	Gallic acid	[21]
**3**	[M-H]^-^	6.87	C_7_H_6_O_4_	153.0187	153.0215	2.8	5	136, 124, 107	Protocatechuic acid	[21]
**4**	[M-H]^-^	8.292	C_34_H_24_O_22_	783.0681	783.0719	−4.15	23	633, 481, 301, 275, 248	Pedunculagin I	[22]
**5**	[M-H]^-^	9.464	C_16_H_18_O_9_	353.0872	353.0909	−8.74	8	191, 179, 135	Chlorogenic acid	[23]
**6**	[M-H]^-^	10.7	C_34_H_24_O_22_	783.0681	783.0744	−7.34	23	481, 301, 275	Pedunculagin II	[22]
**7**	[M-H]^-^	11.6	C_27_H_26_O_19_	653.0996	653.1024	−4.35	15	477, 301	Quercetin-bis-hexuronide	[4]
**8**	[M-H]^-^	11.97	C_27_H_25_O_18_	635.0890	635.0944	−8.51	16	461, 421, 285, 169	Trigalloyl hexose	[22]
**9**	[M-H]^-^	12.5	C_13_H_8_O_8_	291.0146	291.0175	−9.79	10	247, 207, 163	Tachioside	[23]
**10**	[M-H]^-^	13.063	C_7_H_6_O_4_	179.0350	179.0365	−8.43	6	166, 135, 107	Caffeic acid	[21]
**11**	[M-H]^-^	13.15	C_34_H_26_O_22_	785.0837	785.0829	1.78	22	633, 483, 419, 301, 275, 249	Tellimagrandin 1	[23]
**12**	[M-H]^-^	13.649	C_20_H_16_O_13_	463.0518	463.0546	−6.0	13	419, 301	Ellagic acid glucoside	[22]
**13**	[M-H]^-^	14.117	C_13_H_12_O_8_	295.0459	295.0490	−10.33	8	179, 133, 115	Caffeoylmalic acid isomer 1	[24]
**14**	[M-H]^-^	14.988	C_13_H_12_O_8_	295.0459	295.0489	−10.0	8	179, 133, 115	Caffeoylmalic acid isomer 2	[24]
**15**	[M-H]^-^	16.494	C_9_H_10_O_5_	197.0450	197.0513	6.3	5	169, 124	Syringic acid	[21]
**16**	[M-H]^-^	17.9	C_14_H_6_O_8_	300.9999	307.0017	−8.97	12	284, 257, 229	Ellagic acid	[22]
**17**	[M-H]^-^	20.427	C_7_H_6_O_3_	137.0238	137.0288	6.65	5	-	Hydroxybenzoic acid	[21]
**18**	[M-H]^-^	21.013	C_30_H_26_O_13_	593.1295	593.1333	−5.45	18	447, 285, 285	Tiliroside	[21]
**19**	[M-H]^-^	22.01	C_30_H_48_O_6_	503.3378	503.3363	3.0	7	485, 441, 409	Hydroxytormentic acid isomer	[25]
**20**	[M-H]^-^	22.53	C_30_H_48_O_6_	503.3378	503.3416	−8.1	7	485, 441, 295	Hydroxytormentic acid isomer	[25]
**21**	[M-H]^-^	22.72	C_30_H_46_O_7_	517.3171	517.3146	4.78	8	479, 455, 439, 153	Trihydroxyursendioic acid	[25]
**22**	[M-H]^-^	23.11	C_30_H_48_O_5_	487.3429	487.3407	4.5	7	469, 425	Tormentic acid	[25]
**23**	[M-H]^-^	24.21	C_30_H_46_O_5_	485.3272	485.3281	−1.75	8	379, 319, 291, 277, 161	Geumonoid	[25]
**24**	[M-H]^-^	25.03	C_30_H_48_O_4_	471.3480	471.3501	−4.48	7	471, 1029	Pomolic acid	[25]

Ion—the type of ionization (+/−), Rt—retention time, calc.—calculated, exp.—experimental, Δ—error of *m*/*z* measurement, RDB—the number of rings and double bonds.

**Table 2:** The content of selected polyphenols in *G. aleppicum* extract based on the reverse phase high-performance liquid chromatography–diode array detector (RP-HPLC-DAD) analysis.

No.	No. from MS Analysis	Rt (min)	Calc. at λ (nm)	Compound	mg/g of Dry Extract	SD/RSD
1	3	3.41	254	Protocatechuic acid	0.215	0.0/0.0
2	5	4.53	325	Chlorogenic acid	0.016	0.0/0.9
3	-	4.97	254	Kaempferol derivative	0.14	0.0/0.0
4	10	6.144	325	Caffeic acid	0.02	0.0/0.4
5	15	6.152	280	Syringic acid	0.12	0.0/0.1
6	17	6.17	254	Hydroxybenzoic acid	0.261	0.0/0.2
7	13	8.79	325	Caffeic acid derivative 1	0.169	0.0/0.2
8	16	12.24	254	Ellagic acid	2.28	0.0/0.0
9	14	13.54	325	Caffeic acid derivative 2	0.04	0.0/0.4
10	7	16.89	254	Quercetin-derivative	0.05	0.0/0.5

MS—high-performance liquid chromatography coupled with electrospray ionization quadrupole time-of-flight tandem mass spectrometry, Rt—retention time, Calc.—calculated, SD—standard deviations, RSD—relative standard deviation.

**Table 3:** Total polyphenol content, total flavonoid content, and antioxidant activity of *G. aleppicum* extract.

Parameter	Assay	Value	Unit	RSD (%)
Total polyphenol content	Folin–Ciocalteu	131.45 ± 1.84	mg GAE/g	1.40
Total flavonoid content	Pharmacopeial (AlCl_3_)	12.75 ± 0.17	mg QUE/g	1.34
Antioxidant activity	FRAP	3.82 ± 0.07	mmol Fe^2+^/g	1.92
	DPPH	106.61 ± 0.89	mg GAE/g	0.83

RSD—relative standard deviation.

**Table 4:** The minimal inhibitory and minimal bactericidal or fungicidal concentration of *G. aleppicum* extract against chosen reference microorganisms.

Bacteria	Reference Antimicrobial	*G. Aleppicum* Extract			
	MIC (µg/mL)	MIC (mg/mL)	MBC (mg/mL)	MBC/MIC	Effect
*Staphylococcus aureus* ATCC 29213	0.5 *	2	2	1	bactericidal
*Escherichia coli* ATCC 25922	0.004 **	8	8	1	
*Pseudomonas aeruginosa* ATCC 27853	0.5 **	2	8	4	
**Fungi (** **Y** **easts)**		**MIC** (mg/mL)	**MFC** (mg/mL)	**MFC/MIC**	**Effect**
*Candida albicans* ATCC 10231	1 ***	1	8	8	fungistatic
*Candida krusei* ATCC 14243	16 ***	1	8	8	
*Candida parapsilosis* ATCC 22019	0.125 ***	1	8	8	
*Candida tropicalis* ATCC 13803	0.5 ***	0.125	8	64	
*Candida glabrata* ATCC 90030	16 ***	0.125	8	64	
*Candida lusitaniae* ATCC 34449	1 ***	1	4	4	fungicidal
*Candida auris* CDC B11903	>32 ***	8	16	4	

The results of minimal inhibitory concentration and minimal bactericidal or fungicidal concentration are presented as mode based on three independent replications. MIC—minimal inhibitory concentration; MBC—minimal bactericidal concentration; MFC—minimal fungicidal concentration; * vancomycin; ** ciprofloxacin; *** fluconazole.

## Data Availability

The data that support the findings of this study are available from the corresponding author upon reasonable request.

## Supplementary Materials

The following supporting information can be downloaded at https://www.mdpi.com/article/10.3390/molecules30193888/s1, Figure S1: The fingerprints recorded in the positive (above) and negative (below) ion mode during the analysis of *G. aleppicum* extract; Figure S2: Chromatograms of *G. aleppicum* extract obtained with the use of RP-HPLC/PDA. Chromatogram A—measured at 254 nm. Chromatogram B—measured at 325 nm. Chromatogram C—measured at 280 nm. Figure S3: Antiviral potential of *G. aleppicum* extract against Human Coxsackievirus B3; Table S1: MS/MS spectra of compounds identified with the use of HPLC-MS fingerprinting in *Geum aleppicum* Jacq. extract.
