# Supplementary material for: Unraveling the Chemical Composition and Biological Activity of Geum aleppicum Jacq.: Insights from Plants Collected in Kazakhstan
Source: Molecules. 2025 Sep 26;30(19):3888. doi: 10.3390/molecules30193888 (PMC12525716; doi:10.3390/molecules30193888)
Supplement: Supplementary file 1 [file molecules-30-03888-s001.zip › molecules-3865948-supplementary.pdf]

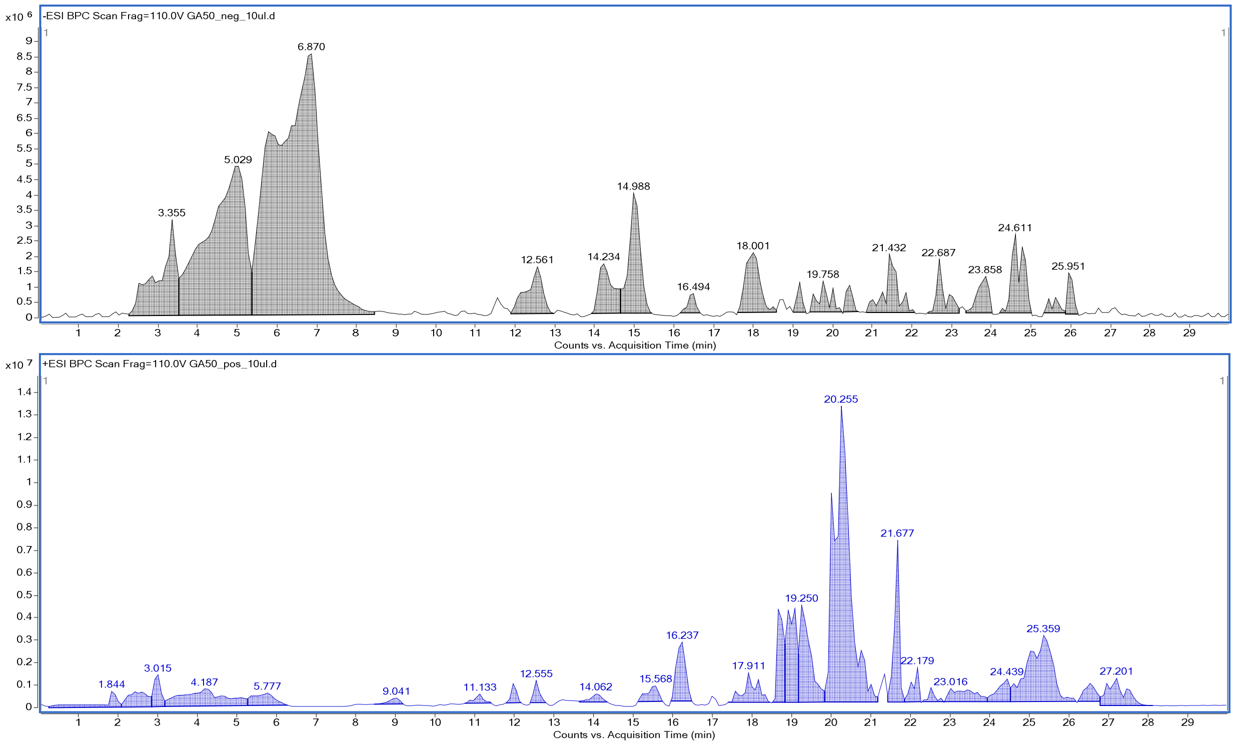

Figure S1. The fingerprints recorded in the negative (above) and positive (below) ion mode during the analysis of *G. aleppicum* extract.

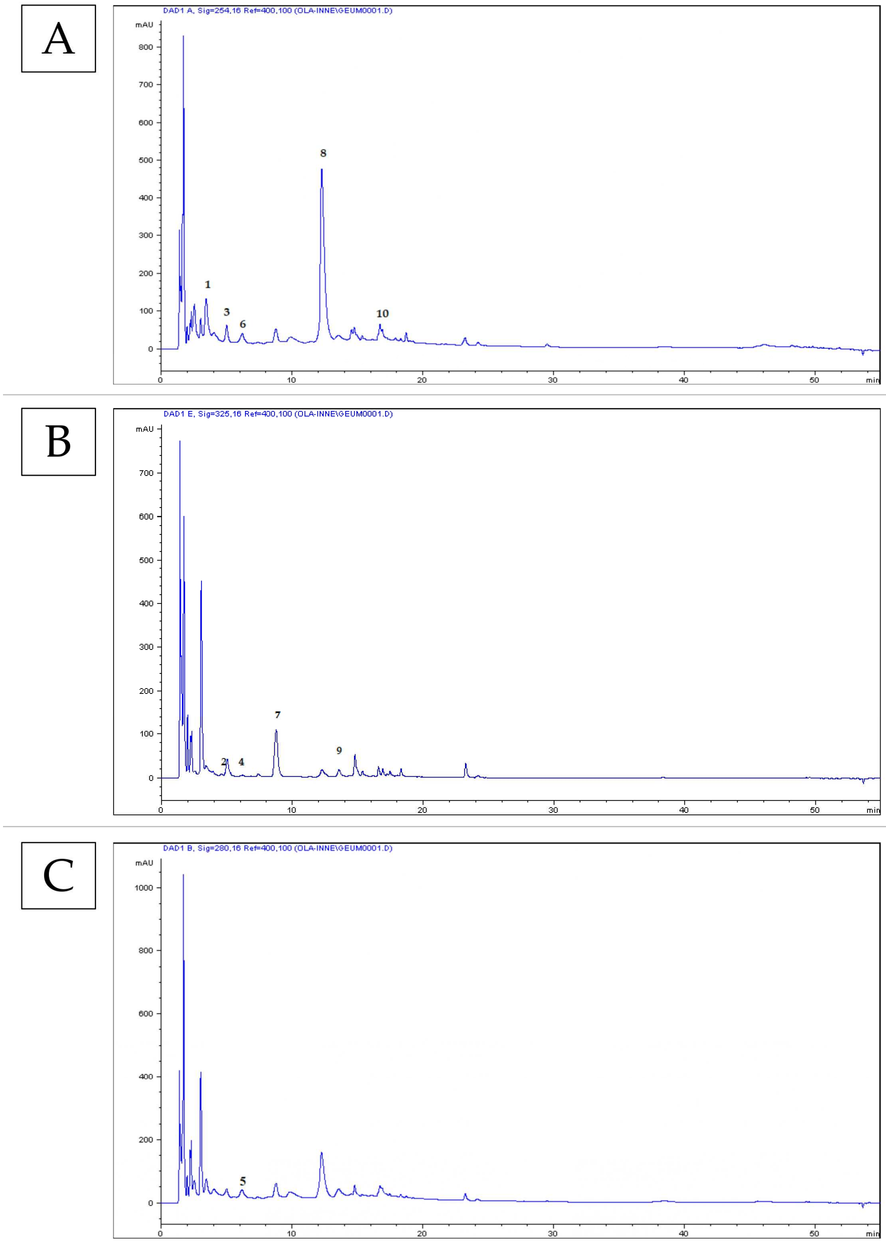

Figure S2. Chromatograms of *G. aleppicum* extract obtained with the use of RP-HPLC/PDA. Chromatogram A – measured at 254 nm. Chromatogram B – measured at 325 nm. Chromatogram C – measured at 280 nm.

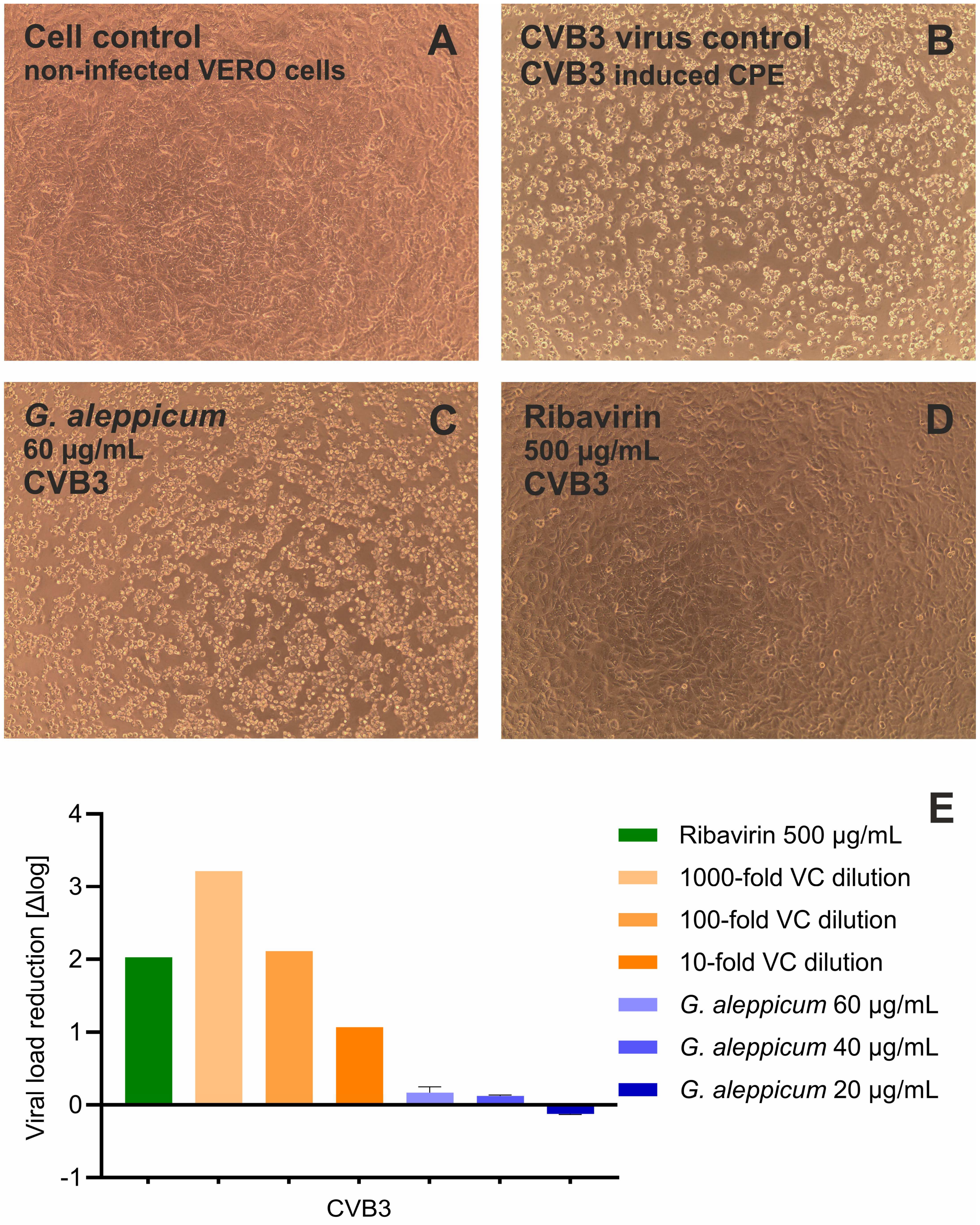

Figure S3. Antiviral potential of *G. aleppicum* extract against Human Coxsackievirus B3. (A) VERO cell monolayer, cell control; (B) CVB3-induced cytopathic effect HHV-1; virus control; (C) influence of *G. aleppicum* 60 µg/mL on CVB3-infected VERO cells; (D) influence of acyclovir 30 µg/mL on CVB3-infected VERO cells; (E) reduction in CVB3 viral load in relation to virus control.

Table S1. MS/MS spectra of compounds identified with the use of HPLC-MS fingerprinting in *G. aleppicum* extract.

<table>
<tr><th>Spectrum</th><th>Proposed compound</th></tr>
<tr><td>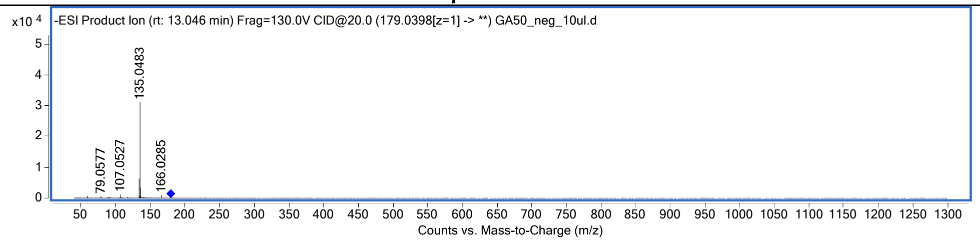
</td><td>Caffeic acid</td></tr>
<tr><td>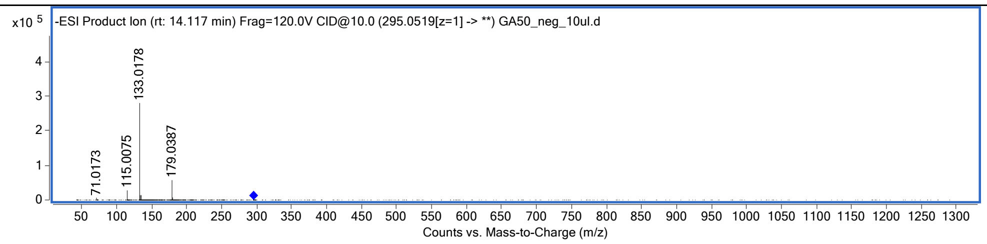
</td><td>Caffeoylmalic acid isomer 1</td></tr>
<tr><td>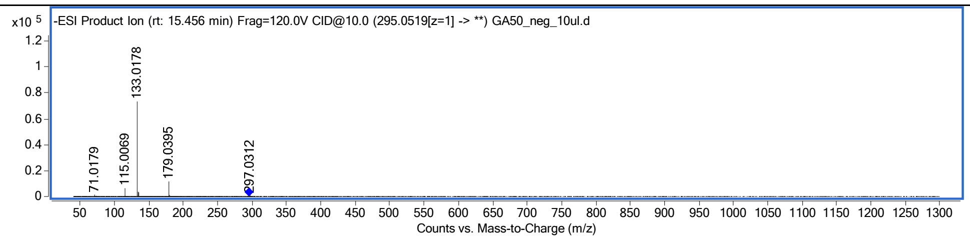
</td><td>Caffeoylmalic acid isomer 2</td></tr>
<tr><td>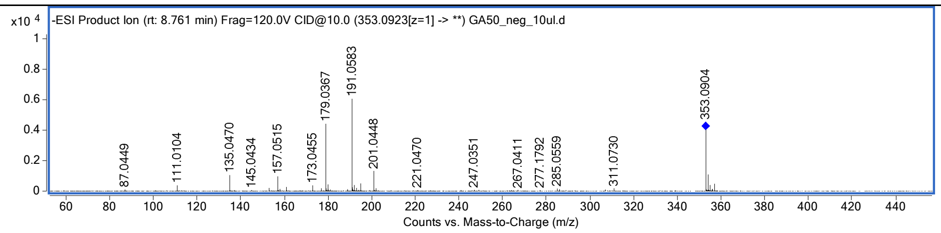
</td><td>Chlorogenic acid</td></tr>
<tr><td>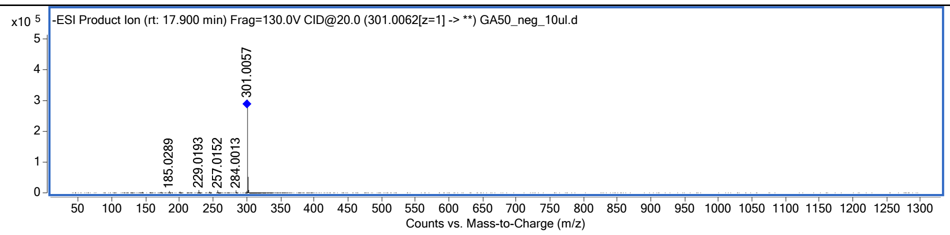
</td><td>Ellagic acid</td></tr>
</table>

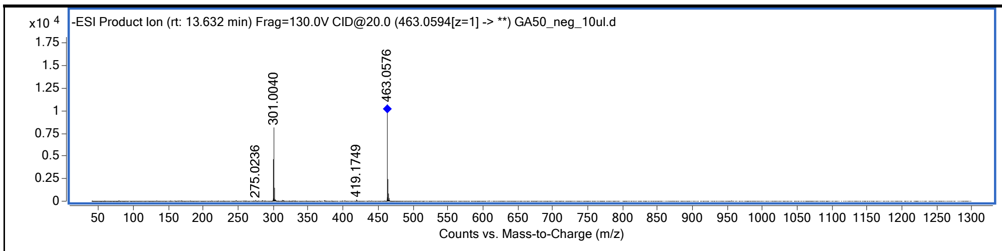	**Ellagic acid glucoside**
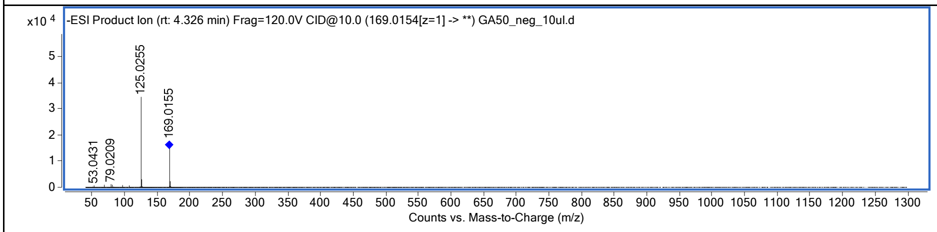	**Gallic acid**
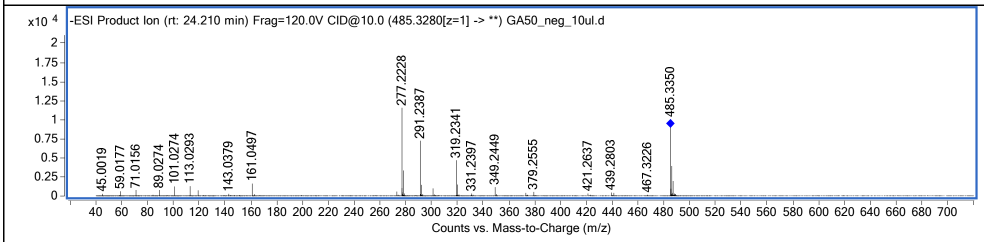	**Geumonoid**
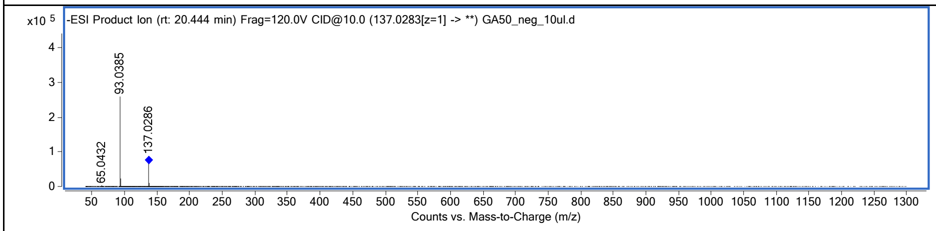	**Hydroxybenzoic acid**
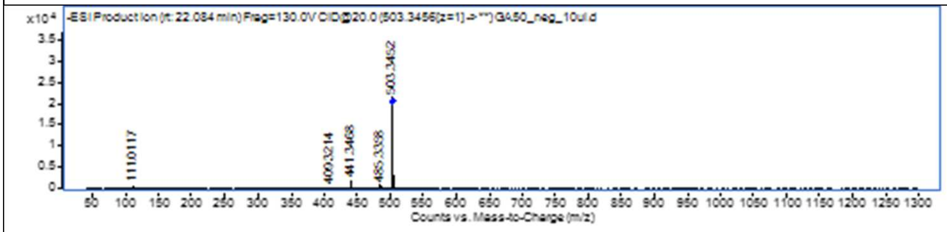	**Hydroxytormentic acid isomer**

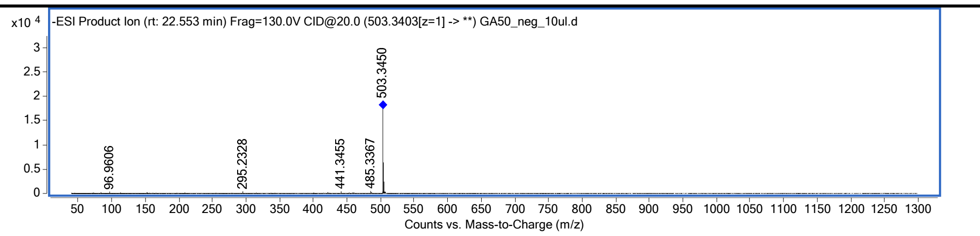

Hydroxytormentic acid isomer

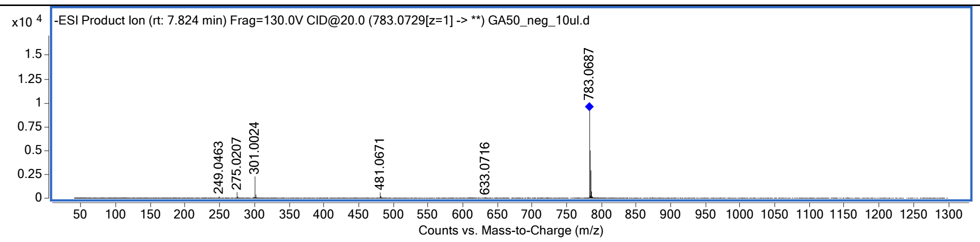

Pedunculagin I

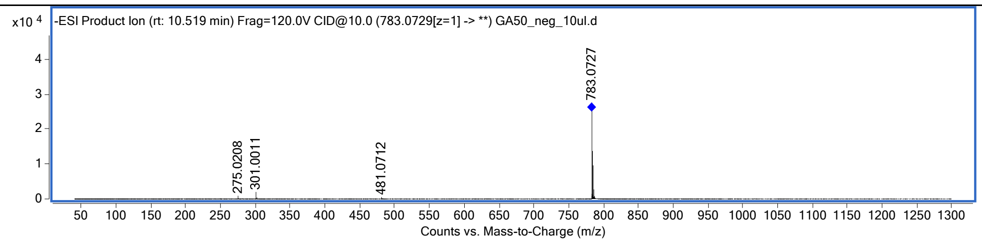

Pedunculagin II

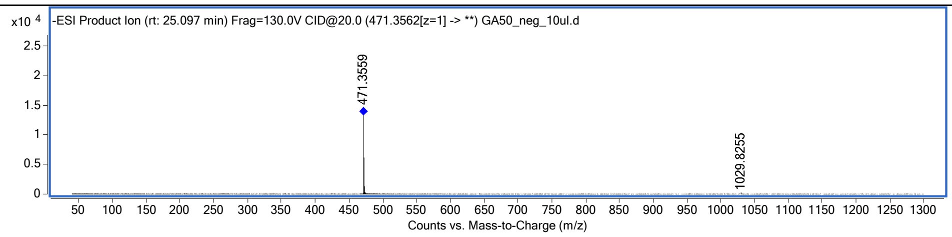

Pomolic acid

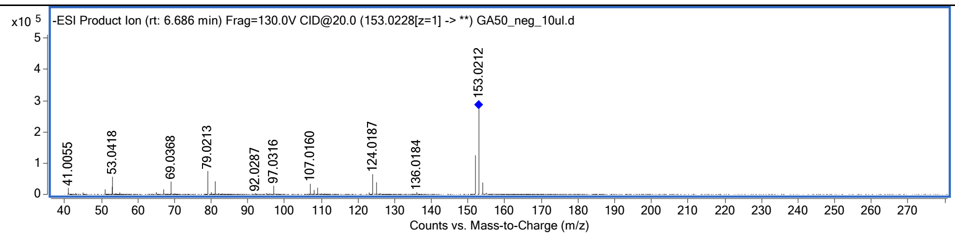

Protocatechuic acid

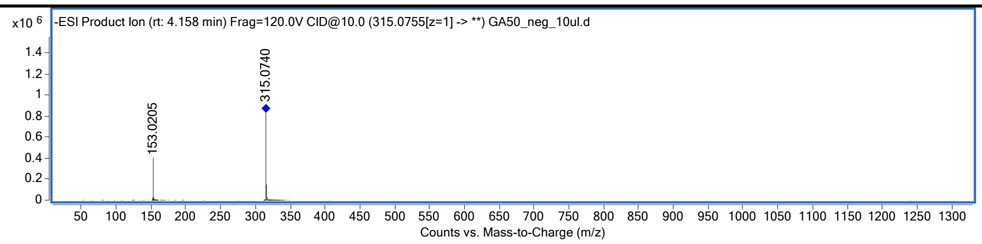

Protocatechuoylglucose

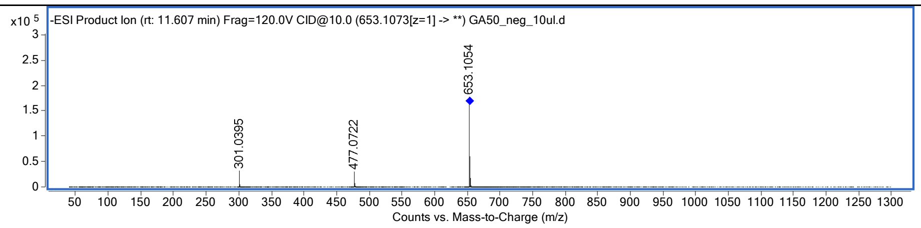

Quercetin bis-hexauronide

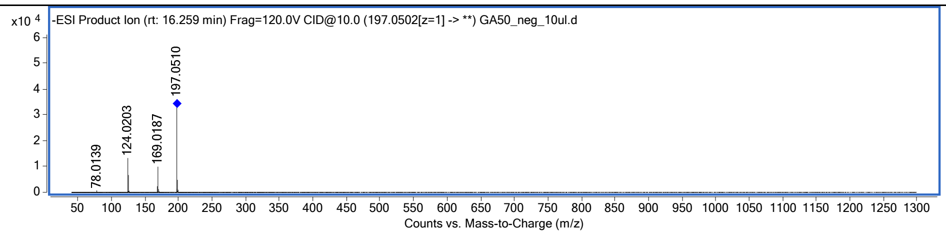

Syringic acid

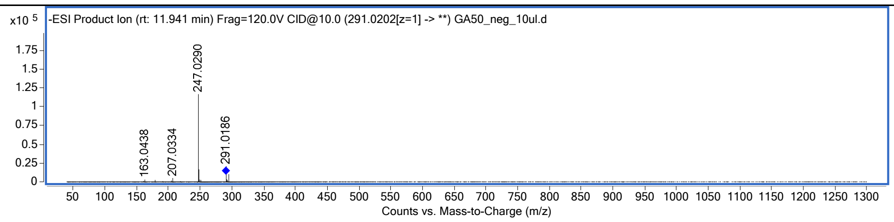

Tachioside

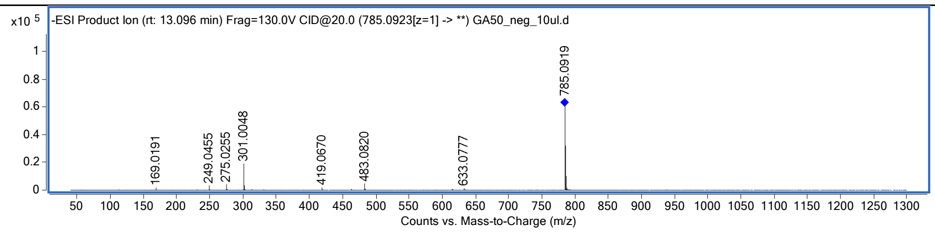

Tellimagrandin 1

<table>
<tr>
<td>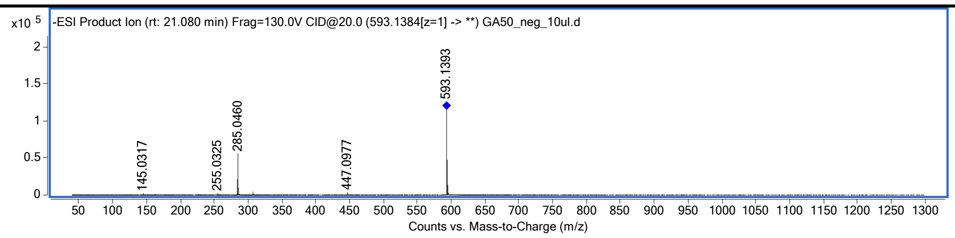
</td>
<td>Tiliroside</td>
</tr>
<tr>
<td>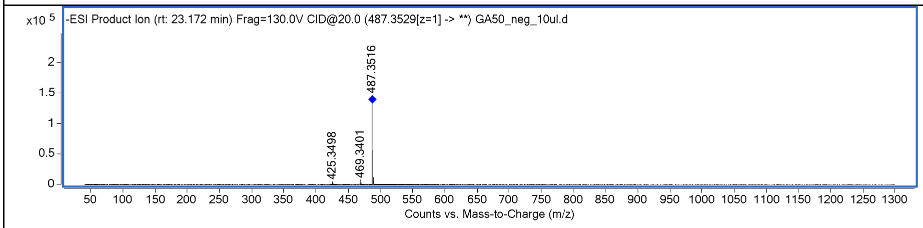
</td>
<td>Tormentic acid</td>
</tr>
<tr>
<td>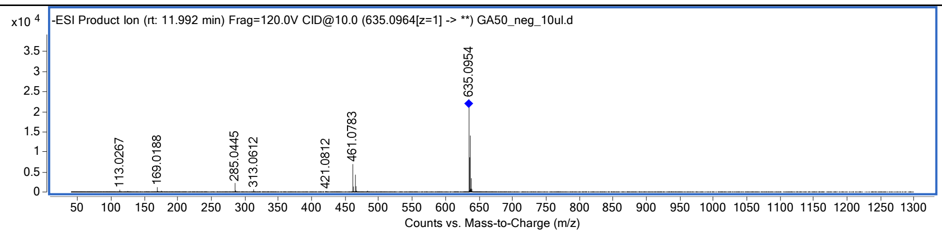
</td>
<td>Trigalloyl hexose</td>
</tr>
<tr>
<td>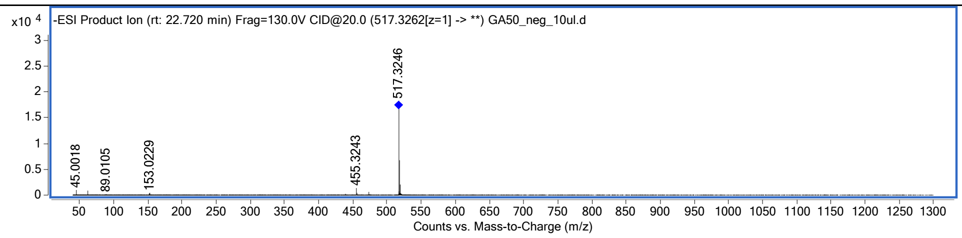
</td>
<td>Trihydroxyursenedioic acid</td>
</tr>
</table>
